# Application of Gold Nanoparticles for Improvement of Electroporation-Assisted Drug Delivery and Bleomycin Electrochemotherapy

**DOI:** 10.3390/pharmaceutics16101278

**Published:** 2024-09-30

**Authors:** Barbora Lekešytė, Eglė Mickevičiūtė, Paulina Malakauskaitė, Anna Szewczyk, Eivina Radzevičiūtė-Valčiukė, Veronika Malyško-Ptašinskė, Augustinas Želvys, Natalija German, Almira Ramanavičienė, Julita Kulbacka, Jurij Novickij, Vitalij Novickij

**Affiliations:** 1 Department of Immunology and Bioelectrochemistry, State Research Institute Centre for Innovative Medicine, 08406 Vilnius, Lithuania; barbora.lekesyte@imcentras.lt (B.L.); egle.mickeviciute@imcentras.lt (E.M.); paulina.malakauskaite@imcentras.lt (P.M.); a.szewczyk@umw.edu.pl (A.S.); eivina.radzeviciute@imcentras.lt (E.R.-V.); augustinas.zelvys@imcentras.lt (A.Ž.); natalija.german@imcentras.lt (N.G.); almira.ramanaviciene@imcentras.lt (A.R.); j.kulbacka@umw.edu.pl (J.K.); 2 Faculty of Electronics, Vilnius Gediminas Technical University, 10105 Vilnius, Lithuania; veronika.malysko-ptasinske@vilniustech.lt (V.M.-P.); jurij.novickij@vilniustech.lt (J.N.); 3 Faculty of Pharmaceutics, Wroclaw Medical University, 50-556 Wroclaw, Poland; 4 NanoTechnas—Center of Nanotechnology and Materials Science, Faculty of Chemistry and Geosciences, Vilnius University, Naugarduko str. 24, 03225 Vilnius, Lithuania

**Keywords:** gold nanoparticles, electrochemotherapy, bleomycin, cancer, pores, electric fields, drug delivery

## Abstract

**Background/Objectives:** Electrochemotherapy (ECT) is a safe and efficient method of targeted drug delivery using pulsed electric fields (PEF), one that is based on the phenomenon of electroporation. However, the problems of electric field homogeneity within a tumor can cause a diminishing of the treatment efficacy, resulting only in partial response to the procedure. This work used gold nano-particles for electric field amplification, introducing the capability to improve available elec-trochemotherapy methods and solve problems associated with field non-homogeneity. **Methods:** We characterized the potential use of gold nanoparticles of 13 nm diameter (AuNPs: 13 nm) in combination with microsecond (0.6–1.5 kV/cm × 100 μs × 8 (1 Hz)) and nanosecond (6 kV/cm × 300–700 ns × 100 (1, 10, 100 kHz and 1 MHz)) electric field pulses. Finally, we tested the most prominent protocols (microsecond and nanosecond) in the context of bleomycin-based electrochemotherapy (*4T1* mammary cancer cell line). **Results:** In the nano-pulse range, the synergistic effects (improved permeabilization and electrotransfer) were profound, with increased pulse burst frequency. Addi-tionally, AuNPs not only reduced the permeabilization thresholds but also affected pore resealing. It was shown that a saturated cytotoxic response with AuNPs can be triggered at significantly lower electric fields and that the AuNPs themselves are non-toxic for the cells either separately or in combination with bleomycin. **Conclusions:** The used electric fields are considered sub-threshold and/or not applicable for electrochemotherapy, however, when combined with AuNPs results in successful ECT, indicating the methodology’s prospective applicability as an anticancer treatment method.

## 1. Introduction

Electrochemotherapy (ECT) is a well-recognized method in cancer treatment, which is based on the transient permeability of cell membrane in electric fields (electroporation), allowing the localized drug delivery and therapy of cancer [1,2]. ECT efficacy depends on pulse characteristics, including waveform, duration, pulse number, or burst delivery frequency, to ensure efficient intracellular molecular transfer [3]. Clinical parametric protocols are based on a series of 100 µs pulses (known as ESOPE protocols) [4]. One of the most significant limitations of ECT is the non-homogeneity of spatial field distribution within the tumor, resulting in tumor regrowth, which is particularly severe in the case of non-invasive electrodes (skin and tumor are heterogeneous) [5,6]. One of the improvements could be the application of ultra-short (nanosecond) pulses [7].

**Figure 1:**
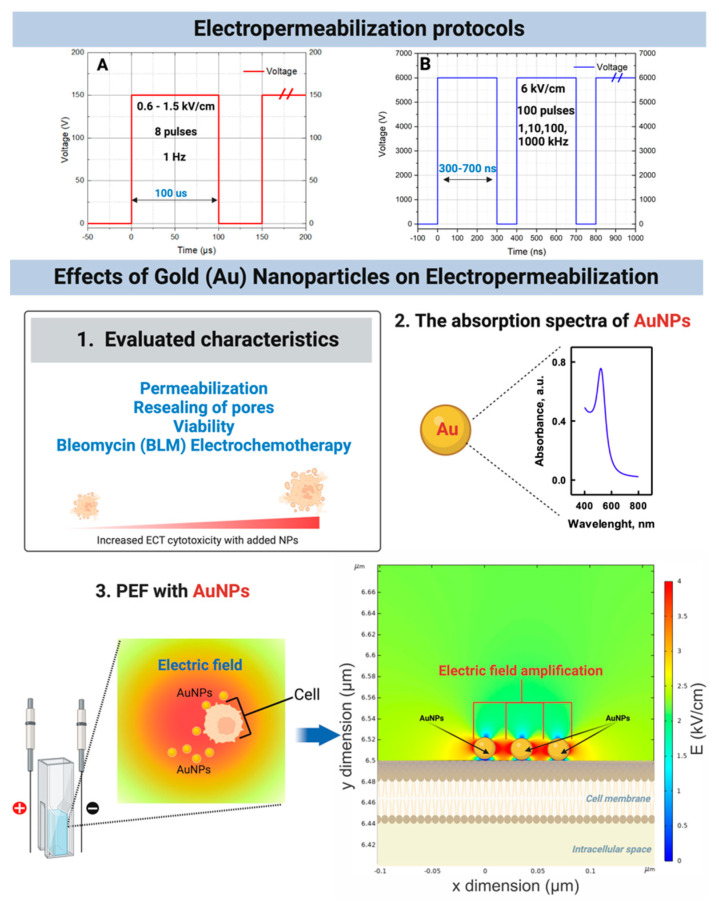
Shows electropermeabilization protocol and effects of Gold (Au) nanoparticles on electropermea-bilization where A and B summaries used pulse parameters and research performed with micro-second or nanosecond protocols respectively, 1—parameters analyzed in the study; 2—absorption spectra of gold nanoparticles involved in the work; 3—the electric field amplification phenomenon with conductive nanoparticles, where electric field distribution is presented for concept visualization purposes only; AuNPs refers to gold nanoparticles, PEF—pulsed electric fields.

Nano-pulses are potentially advantageous for impedance mitigation in heterogeneous tissue in the high-frequency domain [8,9,10]. Also, they allow better controlled Joule heating, reduced muscle contractions, and minimized pain sensation and oxidation due to ROS [8,9,11,12,13]. Recently, it was shown (both in vitro and in vivo) that a residual transmembrane potential (TMP) can be induced, significantly potentiating drug/gene delivery when the pulse repetition frequency is in the MHz range, i.e., the delay between the pulses is shorter than the polarization time of the membrane [14,15,16]. The phenomenon significantly boosted the applicability of nsPEF for drug delivery, which was previously considered sub-optimal due to the lack of electrophoretic components and electrotransfer efficiency compared to ESOPE [17]. Previously, the application of nsPEF was limited to ablation techniques [18] and not applicable to ECT. While it was shown that such an approach (MHz pulse compression) is, in many cases, superior to ESOPE, it is still insufficient to mitigate the PEF’s non-homogeneity completely [19,20]. However, in this work, we optimized the methodology even further, i.e., we characterized the use of conductive gold nanoparticles (AuNPs) to serve as a distributed antennae for PEF amplification in vitro. The summary of the research covered in the paper study is presented in Figure 1.

It has been theoretically predicted that the presence of conductive NPs close to the membrane during PEF treatment results in local enhancement of the electric field [21,22], thus leading to membrane electroporation at lower electric fields [23,24]. Currently, there are less than five in vitro works on the topic [23,24,25,26]; however, all show positive results as proof of concept. Nevertheless, the effects of microsecond pulses are reported mainly in the context of electropermeabilization. In contrast, the efficacy of drug delivery in the context of nanosecond pulses is not covered at all. Therefore, this work aimed to characterize the applicability of the synergistic approach in the context of nanosecond pulses (Figure 1), derive optimal in vitro protocols, and form the first experimental-evidence-based knowledge for further in vivo research. Microsecond pulses were used as a reference to ensure repeatability and consolidation of knowledge. Currently, the effects of conductive NPs in the context of nano-electrochemotherapy are not yet characterized, and the effects of the pulse burst frequency and the interplay with NPs are not covered. At the same time, the potential synergy opens the opportunity to ensure nano-electroporation in significantly lower PEF and a more homogenized treatment.

## 2. Materials and Methods

### 2.1. Electroporation Setup and Parameters

The experimental setup consisted of a 3 kV square-wave high-voltage pulse generator (VilniusTECH, Vilnius, Lithuania) [27] and a commercially available electroporation cuvette with a 1 mm gap between electrodes (Biorad, Hercules, CA, USA). The voltage applied to the cuvette was 0.06–0.6 kV, corresponding to a 0.6–6 kV/cm electric field, respectively. The protocols were used for µsPEF (0.6–1.5 kV/cm × 100 µs × 8, 1 Hz). The nsPEF involved sequences of 100 pulses (6 kV/cm × 300–700 ns × 100) delivered at different frequencies (1, 10, 100, 1000 kHz).

### 2.2. Cell Culture

The RPMI 1640 media supplemented with 10% FBS, glutamine, 25 mM HEPES, 100 U/mL penicillin, and 100 μg/mL streptomycin was used to culture the murine mammary cancer cell line 4T1. The source of all cell culture reagents was Gibco, a division of Thermo Fisher Scientific located in New York, NY, USA. The cells were grown in 5% CO_2_ at 37 °C. On an experimental day, cells were trypsinized (Thermo Fisher Scientific, Grand Island, NY, USA), centrifuged, and resuspended in the electroporation buffer (sucrose 242 mM, Na_2_HPO_4_ 5.5 mM, NaH_2_PO_4_ 3 mM, MgCl_2_ 1.7 mM; pH 7.1) at a concentration of 0.5 × 10^6^ cells/mL. Cells were resuspended at a concentration of 2 × 10^6^ cells/mL in the electroporation solution for the cell permeabilization and viability tests. Prior to electroporation, all samples were incubated for 20 min on ice.

### 2.3. Cell Permeabilization

Yo-Pro1, a green fluorescent stain (Sigma-Aldrich, St. Louis, MO, USA), was used to measure the effectiveness of cell permeabilization. The cells were mixed with Yo-Pro1 at a concentration of 1 μM after being combined (or not) with AuNPs of 13 nm diameter (25 µg/mL) in an electroporation buffer at a concentration of 2 × 10^6^ cells/mL. In order to test the various PEF methods, samples were positioned between the electrodes. After electroporation, 150 μL of phosphate-buffered saline (PBS) was added, followed by 3–10 min incubation at room temperature. The BD Accuri C6 flow cytometer (BD Biosciences, San Jose, CA, USA) was used to analyze the samples. The fluorescence of Yo-Pro1 (491⁄509) was seen in Channel FL1 (533/30 nm BPF).

### 2.4. Viability Assay

Following electroporation, the samples were placed into 96-well flat-bottom plates and incubated on ice for 20 min. Adding growth media to every well achieved a final volume of 200 μL. The wells underwent two PBS washes after a 24 h period. In the case of bleomycin application, concentrations from 0.01 to 5 μg/mL of BLM (Medac, Wedel, Germany) were added separately and in combination with AuNPs, and the viability of the cell was characterized 48 h after treatment. Each well was filled with 150 µL of PBS and 5 µL of cell viability reagent (PrestoBlue, Thermo Fisher Scientific, Grand Island, NY, USA). After giving the cells two hours to incubate, the fluorescence was assessed using a Synergy 2 microplate reader and Gen5 software (PN 5321002, BioTek, Shoreline, WA, USA). Emission was measured at 620/40 nm, and excitation at 540/20 nm.

### 2.5. The Synthesis of 13 nm Gold Nanoparticles

The detailed synthesis procedure and characterization of AuNPs were described earlier [25]. To synthesize AuNPs of 13 nm average diameter, the hydrogen tetrachloroaurate(III) trihydrate (HAuCl_4_·3H_2_O; Carl Roth, GmbH&Co, Karlsruhe, Germany) was reduced by tri-sodium citrate (Penta, Praha, Czech Republic) in the presence of tannic acid (Carl Roth, GmbH&Co, Karlsruhe, Germany) according to the Turkevich method [28,29]. Firstly, 80 mL of 0.0125% [*w*/*v*] HAuCl_4_·3H_2_O solution and 20 mL solution consisting of trisodium citrate (4 mL 1% [*w*/*v*]) and tannic acid (0.025 mL of 1% [*w*/*v*]) were prepared and heated up to +60 °C. After that, both solutions were mixed, heated up to +98 °C with stirring, and kept at this temperature for 3 min to yield a red-wine-colored solution and almost monodisperse AuNPs. Finally, the Erlenmeyer flask containing the 13 nm AuNP colloidal solution was cooled in an ice bath and kept in the refrigerator at +4 °C before use. The diameter of AuNPs evaluated by atomic force microscopy using tapping mode was within the range of 12–16 nm [30]. Additionally, the diameter of spherical AuNPs determined by SEM and TEM was around 12–17 nm [31]. The diameter of synthesized AuNPs determined by the dynamic light scattering technique was 12.96 ± 0.607 nm. Zeta potential measurements showed that 13 nm AuNPs have an average surface charge of −34.2 ± 2.3 mV in the obtained colloidal solution (pH ~6.5). The absorption peak at 521 nm (known as the localized surface plasmon resonance peak) in the absorption spectrum registered by the spectrophotometer Lambda 25 UV/VIS (Perkin Elmer, Connecticut, Shelton, CT, USA) is characteristic for AuNPs of 13 nm diameter.

The initial concentration of gold (based on mass) used for the synthesis of 13 nm AuNPs was 50 µg/mL. The AuNPs solution diluted 1:1 with a cell suspension was used for further studies. The final concentration of gold in the working solution was 25 μg/mL.

### 2.6. Statistical Analysis

One-way analysis of variance (ANOVA; *p* < 0.05) was used to compare the different treatments. When ANOVA indicated a statistically significant result, the Tukey HSD multiple comparison test was used to evaluate the differences (*p* < 0.05 was considered statistically significant). All obtained data were processed in OriginPro software (version 18.0, OriginLab, Northampton, MA, USA). Each data point was obtained from at least three independent experiments, and the results are represented as mean ± standard deviation.

## 3. Results

### 3.1. Effects of BLM and AuNPs on Cell Viability

**Figure 2:**
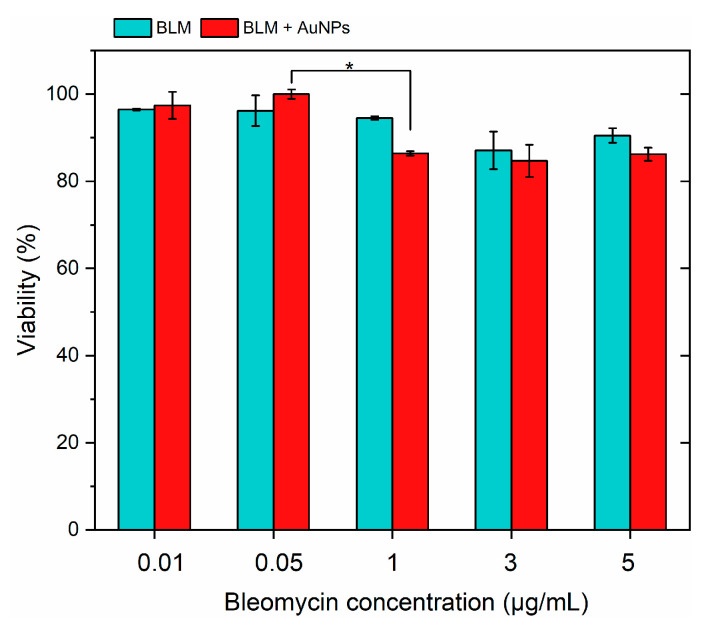
The effects of bleomycin (BLM) separately and in combination with gold nanoparticles (AuNPs) on the viability of the 4T1 cell line 48 h after treatment. Asterisk (*) corresponds to a statistically significant difference (*p* < 0.05).

Electrochemotherapy is based on the application of cytotoxic compounds using doses significantly lower than the ones used in chemotherapy. At the same time, the toxicity of the treatment is boosted by transient permeabilization of the cell plasma membrane and targeted intracellular delivery of the drugs. Therefore, it is essential to select a drug concentration that is not toxic to the cells. Also, since the proposed methodology also involved application of AuNPs, the NPs in combination with BLM should also must be predominantly non-toxic [16,32]. Therefore, firstly, we characterized the effects of BLM at various concentrations on the viability of *4T1* cancer cell lines separately and in combination with AuNPs. The results are summarized in Figure 2.

It can be seen that AuNPs were non-toxic to the cells, while BLM had a minor effect (<20%) on cell viability in the whole range of tested concentrations (0.01–5 μg/mL). Based on the acquired results, the 1 μg/mL BLM concentration was used further in the study.

### 3.2. Membrane Permeabilization Using Nano- and Microsecond Electric Fields

**Figure 3:**
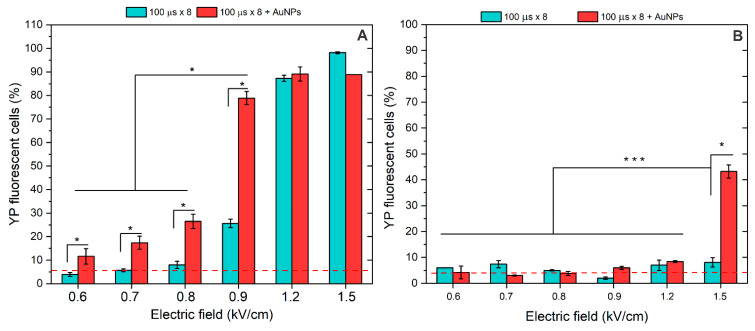
The effects of pulsed electric fields (100 μs × 8) on (**A**) cell plasma membrane permeabilization to Yo-Pro-1 and (**B**) cell plasma membrane resealing after 30 min, separately and in combination with AuNPs. Asterisk (*, ***) corresponds to a statistically significant (*p* < 0.05 and *p* < 0.001) difference, respectively. Red dash corresponds to untreated control samples.

Further, we characterized the effects of pulsed electric fields on membrane permeabilization separately and in combination with AuNPs. Figure 3 summarizes the effects of microsecond (100 μs) PEF.

The standard ESOPE protocols (100 μs × 8) were tested in a broad range of amplitudes (0.6–1.5 kV/cm). As shown in Figure 3A, without AuNPs, high permeabilization was ensured only with 1.2 and 1.5 kV/cm PEF amplitude, which is in agreement with established knowledge. The 0.9 kV/cm protocol induced <30% cell membrane permeabilization, thus being not applicable for ECT. However, when AuNPs were involved, a definitive improvement in cell permeabilization was achieved across the whole range of tested amplitudes. As a result, the 0.9 kV/cm PEF, when combined with AuNPs, triggered >75% cell permeabilization, which was more than a 40% improvement.

The experiments were followed by pore resealing analysis when the fluorescent dye was added 30 min after electroporation. The results are summarized in Figure 3B. It can be seen that in most cases, electroporation was reversible (i.e., the permeability of Yo-Pro-1 is comparable (*p* > 0.05) with the PEF untreated control). The only exception was the 1.5 kV/cm protocol, which, when combined with AuNPs, irreversibly resulted in >40% of cells being permeabilized.

**Figure 4:**
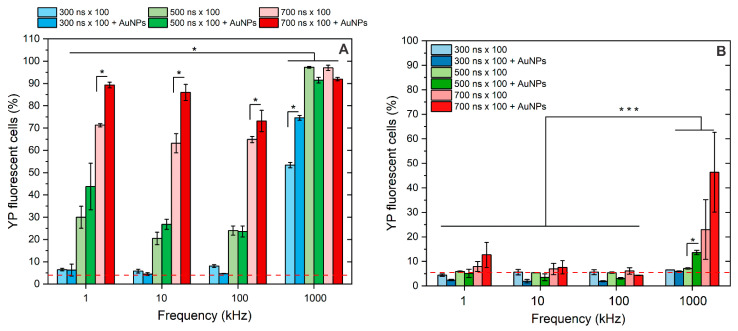
The effects of pulsed electric fields (300–700 ns × 100) on (**A**) cell plasma membrane permeabilization to Yo-Pro-1 and (**B**) cell plasma membrane resealing after 30 min, separately and in combination with AuNPs. Asterisk (*, ***) corresponds to a statistically significant (*p* < 0.05 and *p* < 0.001) difference, respectively. Red dash corresponds to untreated control samples.

A similar analysis was performed for nanosecond sequences (300–700 ns), and the results are presented in Figure 4.

As expected, the longer the pulse duration, the higher the permeabilization of the cell plasma membrane achieved. In the 1–100 kHz range, the 6 kV/cm × 300 ns × 100 pulses did not trigger significant permeabilization, 500 ns × 100 bursts triggered low sub-30% permeabilization, and 700 ns × 100 pulses induced ~60–70% permeabilization of cells. On average, the permeabilization triggered by 1 kHz pulses was higher than that triggered by 10 and 100 kHz bursts. However, the differences (5–20%) were insignificant in the applied context for real applications (e.g., electrochemotherapy). Thus, protocols can be used interchangeably depending on available infrastructure.

When 1 MHz pulses were used, the residual TMP accumulation was triggered. Therefore, the permeabilization rate was dramatically increased using the same pulsing protocols. Even the 300 ns × 100 pulses made more than 50% of cells permeabilized.

At the same time, the 500 ns and 700 ns pulses triggered saturated permeabilization (>95%), which was significantly higher than the effect of 1–100 kHz bursts. The impact of AuNPs was similar to microsecond pulses, i.e., the permeabilization threshold was reduced, and a higher number of YP fluorescent cells was observed (unless saturated permeabilization was already triggered by PEF alone). This was true for all the protocols involved in the study, except the lower frequency (1–100 kHz) and the 300 ns x 100 sequence. In this frequency range, no amplification of the effect was triggered, while the phenomenon was still present in the case of a 1 MHz burst.

Finally, the resealing of the pores following nsPEF treatment was characterized, and the results are summarized in Figure 4B. It can be seen that all of the 1–100 kHz protocols triggered predominantly reversible electroporation, while in the case of 1 MHz sequences, the 700 ns × 100 protocol resulted in a higher fraction of irreversibly permeabilized cells, which was further amplified if AuNPs were added.

**Figure 5:**
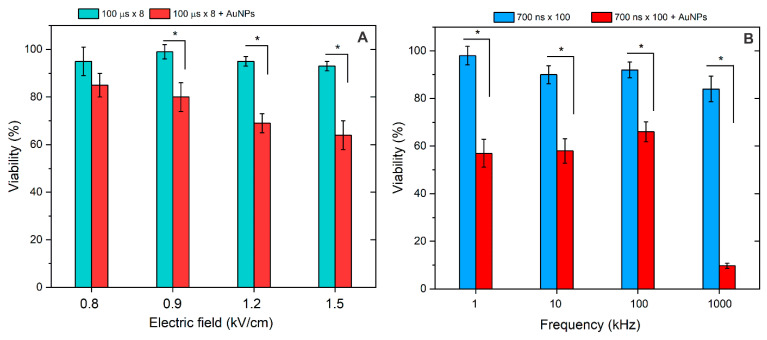
The effects of pulsed electric fields on cell viability (24 h after treatment), with (**A**) 100 μs × 8 protocols and (**B**) 700 ns × 100 protocols, separately and in combination with AuNPs. Asterisk (*) corresponds to a statistically significant (*p* < 0.05) difference.

Based on the results, the study was further limited to 700 ns protocols since ECT protocols resulting in high permeabilization were applicable. Cell viability following the treatment was analyzed. The results are summarized in Figure 5.

As can be seen in Figure 5, both ESOPE and nsPEF protocols resulted predominantly in reversible electroporation (>80% viability), which is in agreement with the pore resealing data. In the case of nsPEF, as expected, the 1 MHz burst, on average, resulted in the highest drop in cell viability. Due to the reduction of permeabilization thresholds and higher permeabilization, the viability dropped even further when the AuNPs were added. The scaling was dose-dependent (scales with amplitude) in the case of ESOPE protocols (Figure 5A). In the case of nsPEF, frequency was a minor parameter in the 1–100 kHz range with no significant effect on viability, which was also predicted by the permeabilization data. Only the 1 MHz protocol produced predominantly irreversible electroporation when adding AuNPs.

**Figure 6:**
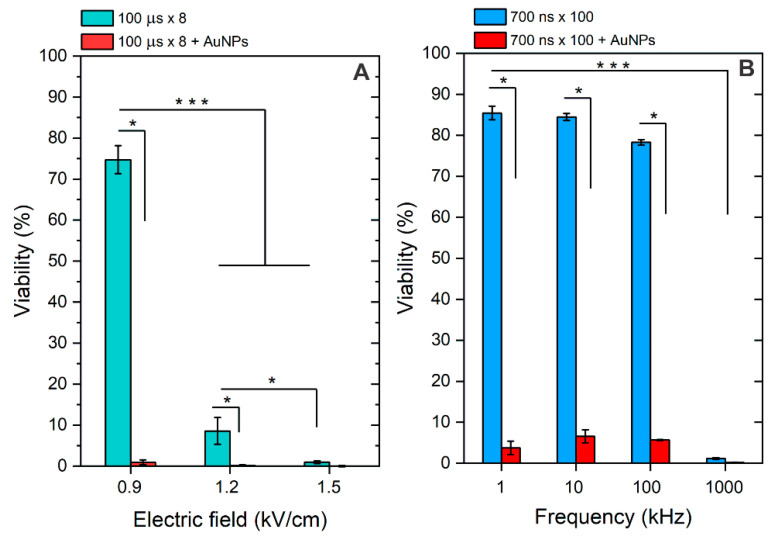
The effects of BLM-based electrochemotherapy on cell viability (48 h after treatment), with (**A**) 100 μs × 8 protocols and (**B**) 700 ns × 100 protocols, separately and in combination with AuNPs. Asterisk (*, ***) corresponds to a statistically significant (*p* < 0.05 and *p* < 0.001) difference, respectively.

Finally, the protocols were tested in the context of bleomycin-based electrochemotherapy, where 1 μg/mL of BLM was added separately and in combination with AuNPs. Cell viability was characterized 48 h after treatment. The results are summarized in Figure 6.

As can be seen in Figure 6A, the 1.2 and 1.5 kV/cm protocols resulted in successful ECT in vitro, which is in agreement with established knowledge [28], while 0.9 kV/cm was inefficient. The result was influenced by low permeabilization when this protocol was applied (Figure 3A). However, when the AuNPs were added, the 0.9 kV/cm became applicable for ECT due to a significant potentiation of the effect (reduction of the permeabilization threshold). In the case of nsPEF, 1–100 kHz protocols were inefficient due to insufficient electrophoretic components, which is also an expected result influencing the lack of applicability of nsPEF protocols in ECT. However, when AuNPs were introduced, all the described protocols triggered excellent cytotoxicity, enabling their applicability for drug delivery. In the case of the 1 MHz protocol, the pulses triggered saturated ECT in vitro. Thus, the effect of AuNPs is not even required (partly irreversible electroporation).

## 4. Discussion

Electrochemotherapy (ECT) is an effective method for targeted drug delivery using pulsed electric fields. However, facing issues with electric field homogeneity in tumors can reduce treatment efficacy [33]. This study investigated the use of gold nanoparticles in order to amplify an electric field in close proximity to the cell membrane. The in vitro proof of concept in the context of ECT (tested in our work) supports the idea of using conductive nanoparticles for improvement (amplification) of the electric field inside the tumor in regions where the highest non-homogeneity of electric field is expected.

It was shown that AuNPs lower permeabilization thresholds and influence pore resealing (even irreversible electroporation can be triggered using parameters associated with reversible electroporation), while a saturated cytotoxic response (with bleomycin) can be triggered with lower electric fields than those typically required for ECT. At the same time, the nanoparticles (by themselves) remain non-toxic to cells, suggesting a promising approach for improving electrochemotherapy techniques.

The effectiveness of electrochemotherapy (ECT) is influenced by various pulse characteristics, including waveform, duration, number of pulses, and burst delivery frequency, which are crucial for facilitating efficient intracellular molecular transfer. Clinical protocols typically use a series of 100 µs pulses called ESOPE protocols [33,34]. In our work, we also tested the applicability of nanosecond pulses and characterized the effects of pulse repetition frequency on ECT and synergy with AuNPs. We have shown that microsecond PEFs already trigger excellent ECT in vitro at 0.9 kV/cm, which is promising for further use in vivo following the injection of NPs in regions of tumors where a reduction of PEF amplitude is expected. In the case of nanosecond pulses, it was shown that pulse repetition frequency can be a flexible parameter for control of cell membrane permeabilization without altering the burst energy. High-frequency (1 MHz) pulses can be used to increase the efficiency of ECT due to the residual TMP accumulation phenomenon (Figure 6B, 1 MHz protocol). Importantly, it was crucial to confirm if the nanoparticles have a synergy with nanosecond bursts, which we successfully did. Independently on the frequency, there is a statistically significant improvement in permeabilization and ECT efficiency when AuNPs are involved. Basically, it is possible to use AuNPs and improve the efficacy of molecular transfer (in this case, BLM) while still staying in the reversible electroporation range (Figure 6B, 1–100 kHz frequency range).

Modeling gold nanoparticles (AuNPs) using computers can offer valuable insights into how electric fields can be altered due to the unique optical and electrical properties of these nanostructures. Previously published work described how computational techniques are utilized to predict and optimize field enhancements around AuNPs [35], demonstrated the role of nanoparticle aggregation on electric field modification through computational models [36], and highlighted how shape and composition influence the plasmonic properties and electric fields around AuNPs [37]. The computer models provide a deep understanding of how electric fields can be manipulated around AuNPs, offering pathways for designing advanced nanoparticles for sensing, imaging, and energy applications. Therefore, computer modeling could potentially be used to model our experimental design in order to optimize the described methodology, but for now, this is a matter of future work.

Currently, there are only several in vitro works on the topic [23,24,25,26,38], all showing positive results as a proof of concept to use conductive NPs with electroporation. This study is the first to characterize the potential applicability in the context of ECT and nanosecond electric field. However, the effects of Z-potential, or the role of different metals (e.g., Fe or Pt), optimal NPs concentration, etc., are not yet known. The optimal NP diameter is also not evident, and due to the novelty of the approach, there is not a single in vivo work supporting the feasibility of the methodology. Based on the results of our and other groups, it is evident that further research is required, which potentially will result in a new modality of ECT involving conductive nanoparticles.

It should be highlighted that the safety of PEF has already been approved in clinics [39], while conductive NPs are routinely used as nanocarriers [40] and/or in the context of hyperthermia with magnetic fields [41]; therefore, there are no biocompatibility or other issues to ensure further translational research.

## 5. Conclusions

In conclusion, this study highlights the effectiveness of electrochemotherapy in administering cytotoxic compounds at lower pulsed electric fields, which are typically associated with the procedure. The presence of AuNPs significantly improved cell membrane permeabilization. This research further supports the optimization of electrochemotherapy protocols by introducing synergistic techniques to improve permeabilization, paving the way for improved therapeutic outcomes in cancer treatment. We speculate that the capability to inject conductive nanoparticles in regions of high interest (e.g., where the electric field is non-homogeneous), or in order to ensure higher susceptibility of the targeted, localized tissue volume, will play a significant role in ensuring a full and well-controlled antitumor response to PEF-based treatment. While we have shown that it is possible to use nanoparticles both with microsecond and nanosecond pulses, further studies will be essential to optimize the proposed technique and to evaluate the long-term effects in clinical applications. The optimization in terms of NPs size, zeta-potential, and type of metal used should be performed in the future. The feasibility of using nano-structures (i.e., rods, stars, etc.) should also be characterized since the electric field amplification is expected to depend on the form factor.

## Data Availability

Data are available from the corresponding author, B.L., on request.

## References

[1] Gehl J. (2015). Drug and Gene Electrotransfer in Cancer Therapy. Somatic Genome Manipulation: Advances, Methods, and Applications.

[2] Miklavčič D., Mali B., Kos B., Heller R., Serša G. (2014). Electrochemotherapy: From the Drawing Board into Medical Practice. Biomed. Eng. Online.

[3] Pucihar G., Krmelj J., Reberšek M., Napotnik T.B., Miklavčič D. (2011). Equivalent Pulse Parameters for Electroporation. IEEE Trans. Biomed. Eng..

[4] Marty M., Sersa G., Garbay J.R., Gehl J., Collins C.G., Snoj M., Billard V., Geertsen P.F., Larkin J.O., Miklavcic D. (2006). Electrochemotherapy—An Easy, Highly Effective and Safe Treatment of Cutaneous and Subcutaneous Metastases: Results of ESOPE (European Standard Operating Procedures of Electrochemotherapy) Study. Eur. J. Cancer Suppl..

[5] Ivorra A., Al-Sakere B., Rubinsky B., Mir L.M. (2008). Use of Conductive Gels for Electric Field Homogenization Increases the Antitumor Efficacy of Electroporation Therapies. Phys. Med. Biol..

[6] Edd J.F., Davalos R.V. (2007). Mathematical Modeling of Irreversible Electroporation for Treatment Planning. Technol. Cancer Res. Treat..

[7] Schoenbach K.H., Hargrave B., Joshi R.P., Kolb J.F., Nuccitelli R., Osgood C., Pakhomov A., Stacey M., Swanson R.J., White J.A. (2007). Bioelectric Effects of Intense Nanosecond Pulses. IEEE Trans. Dielectr. Electr. Insul..

[8] Bhonsle S.P., Arena C.B., Sweeney D.C., Davalos R.V. (2015). Mitigation of Impedance Changes Due to Electroporation Therapy Using Bursts of High-Frequency Bipolar Pulses. Biomed. Eng. Online.

[9] Yao C., Dong S., Zhao Y., Lv Y., Liu H., Gong L., Ma J., Wang H., Sun Y. (2017). Bipolar Microsecond Pulses and Insulated Needle Electrodes for Reducing Muscle Contractions during Irreversible Electroporation. IEEE Trans. Biomed. Eng..

[10] Novickij V., Balevičiute A., Malysko V., Želvys A., Radzevičiute E., Kos B., Zinkeviciene A., Miklavčič D., Novickij J., Girkontaite I. (2022). Effects of Time Delay Between Unipolar Pulses in High Frequency Nano-Electrochemotherapy. IEEE Trans. Biomed. Eng..

[11] Miklavčič D., Pucihar G., Pavlovec M., Ribarič S., Mali M., MačEk-Lebar A., Petkovšek M., Nastran J., Kranjc S., Čemažar M. (2005). The Effect of High Frequency Electric Pulses on Muscle Contractions and Antitumor Efficiency in Vivo for a Potential Use in Clinical Electrochemotherapy. Bioelectrochemistry.

[12] Mahnič-Kalamiza S., Miklavčič D. (2020). Scratching the Electrode Surface: Insights into a High-Voltage Pulsed-Field Application from in Vitro & in Silico Studies in Indifferent Fluid. Electrochim. Acta.

[13] Sano M.B., Fan R.E., Cheng K., Saenz Y., Sonn G.A., Hwang G.L., Xing L. (2018). Reduction of Muscle Contractions during Irreversible Electroporation Therapy Using High-Frequency Bursts of Alternating Polarity Pulses: A Laboratory Investigation in an Ex Vivo Swine Model. J. Vasc. Interv. Radiol..

[14] Novickij V., Ruzgys P., Grainys A., Šatkauskas S. (2018). High Frequency Electroporation Efficiency Is under Control of Membrane Capacitive Charging and Voltage Potential Relaxation. Bioelectrochemistry.

[15] Semenov I., Casciola M., Ibey B.L., Xiao S., Pakhomov A.G. (2018). Electropermeabilization of Cells by Closely Spaced Paired Nanosecond-Range Pulses. Bioelectrochemistry.

[16] Radzevičiūtė E., Malyško-Ptašinskė V., Kulbacka J., Rembiałkowska N., Novickij J., Girkontaitė I., Novickij V. (2022). Nanosecond Electrochemotherapy Using Bleomycin or Doxorubicin: Influence of Pulse Amplitude, Duration and Burst Frequency. Bioelectrochemistry.

[17] Vižintin A., Marković S., Ščančar J., Miklavčič D. (2021). Electroporation with Nanosecond Pulses and Bleomycin or Cisplatin Results in Efficient Cell Kill and Low Metal Release from Electrodes. Bioelectrochemistry.

[18] Nuccitelli R., Pliquett U., Chen X., Ford W., James Swanson R., Beebe S.J., Kolb J.F., Schoenbach K.H. (2006). Nanosecond Pulsed Electric Fields Cause Melanomas to Self-Destruct. Biochem. Biophys. Res. Commun..

[19] Radzeviciute-Valciuke E., Zelvys A., Mickeviciute E., Gecaite J., Zinkeviciene A., Malysko-Ptasinske V., Kaseta V., Novickij J., Ivaskiene T., Novickij V. (2023). Calcium Electrochemotherapy for Tumor Eradication and the Potential of High-Frequency Nanosecond Protocols. Pharmaceuticals.

[20] Balevičiūtė A., Radzevičiūtė E., Želvys A., Malyško-Ptašinskė V., Novickij J., Zinkevičienė A., Kašėta V., Novickij V., Girkontaitė I. (2022). High-Frequency Nanosecond Bleomycin Electrochemotherapy and Its Effects on Changes in the Immune System and Survival. Cancers.

[21] Lekner J. (2014). Electroporation in Cancer Therapy without Insertion of Electrodes. Phys. Med. Biol..

[22] Qiu H., Joshi R.P., Pradhan A. (2014). Simulation of Nanoparticle Based Enhancement of Cellular Electroporation for Biomedical Applications. J. Appl. Phys..

[23] Rezaee Z., Yadollahpour A., Bayati V., Dehbashi F.N. (2017). Gold Nanoparticles and Electroporation Impose Both Separate and Synergistic Radiosensitizing Effects in HT-29 Tumor Cells: An in Vitro Study. Int. J. Nanomed..

[24] Miklavcic D., Novickij V., Kranjc M., Polajzer T., Haberl Meglic S., Batista Napotnik T., Romih R., Lisjak D. (2020). Contactless Electroporation Induced by High Intensity Pulsed Electromagnetic Fields via Distributed Nanoelectrodes. Bioelectrochemistry.

[25] Radzeviciute-Valciuke E., Gecaite J., Zelvys A., Zinkeviciene A., Zalneravicius R., Malysko-Ptasinske V., Nemeikaite-Ceniene A., Kaseta V., German N., Novickij J. (2023). Improving NonViral Gene Delivery Using MHz Bursts of Nanosecond Pulses and Gold Nanoparticles for Electric Field Amplification. Pharmaceutics.

[26] Ghorbel A., Andre F.M., Mir L.M., Garcia-Sanchez T. (2021). Electrophoresis-Assisted Accumulation of Conductive Nanoparticles for the Enhancement of Cell Electropermeabilization. Bioelectrochemistry.

[27] Novickij V., Staigvila G., Murauskas A., Rembialkowska N., Kulbacka J., Novickij J. (2022). High Frequency Bipolar Electroporator with Double-Crowbar Circuit for Load-Independent Forming of Nanosecond Pulses. Appl. Sci..

[28] Mühlpfordt H. (1982). The Preparation of Colloidal Gold Particles Using Tannic Acid as an Additional Reducing Agent. Experientia.

[29] Ramanaviciene A., Nastajute G., Snitka V., Kausaite A., German N., Barauskas-Memenas D., Ramanavicius A. (2009). Spectrophotometric Evaluation of Gold Nanoparticles as Red-Ox Mediator for Glucose Oxidase. Sens. Actuators B Chem..

[30] German N., Ramanavicius A., Voronovic J., Ramanaviciene A. (2012). Glucose Biosensor Based on Glucose Oxidase and Gold Nanoparticles of Different Sizes Covered by Polypyrrole Layer. Colloids Surfaces A Physicochem. Eng. Asp..

[31] Romaskevic T., Sedlevicius M., Budriene S., Ramanavicius A., Ryskevic N., Miasojedovas S., Ramanaviciene A. (2011). Assembly and Characterization of Polyurethane-Gold Nanoparticle Conjugates. Macromol. Chem. Phys..

[32] Niżnik Ł., Noga M., Kobylarz D., Frydrych A., Krośniak A., Kapka-Skrzypczak L., Jurowski K. (2024). Gold Nanoparticles (AuNPs)-Toxicity, Safety and Green Synthesis: A Critical Review. Int. J. Mol. Sci..

[33] Condello M., D’Avack G., Spugnini E.P., Meschini S. (2022). Electrochemotherapy: An Alternative Strategy for Improving Therapy in Drug-Resistant SOLID Tumors. Cancers.

[34] Romeo S., Sannino A., Scarfì M.R., Vernier P.T., Cadossi R., Gehl J., Zeni O. (2018). ESOPE-Equivalent Pulsing Protocols for Calcium Electroporation: An In Vitro Optimization Study on 2 Cancer Cell Models. Technol. Cancer Res. Treat..

[35] Maier A.S., Informatika T., Teknik F., Indonesia U.K. (2007). Plasmonics: Fundamentals and Applications.

[36] Prodan E., Nordlander P. (2004). Plasmon Hybridization in Spherical Nanoparticles. J. Chem. Phys..

[37] Jain P.K., Lee K.S., El-Sayed I.H., El-Sayed M.A. (2006). Calculated Absorption and Scattering Properties of Gold Nanoparticles of Different Size, Shape, and Composition:  Applications in Biological Imaging and Biomedicine. J. Phys. Chem. B.

[38] Zu Y., Huang S., Liao W.-C., Lu Y., Wang S. (2014). Gold Nanoparticles Enhanced Electroporation for Mammalian Cell Transfection. J. Biomed. Nanotechnol..

[39] Yarmush M.L., Golberg A., Serša G., Kotnik T., Miklavčič D. (2014). Electroporation-Based Technologies for Medicine: Principles, Applications, and Challenges. Annu. Rev. Biomed. Eng..

[40] Tietze R., Zaloga J., Unterweger H., Lyer S., Friedrich R.P., Janko C., Pöttler M., Dürr S., Alexiou C. (2015). Magnetic Nanoparticle-Based Drug Delivery for Cancer Therapy. Biochem. Biophys. Res. Commun..

[41] Wahajuddin, Arora S. (2012). Superparamagnetic Iron Oxide Nanoparticles: Magnetic Nanoplatforms as Drug Carriers. Int. J. Nanomed..

